# pcrEfficiency: a Web tool for PCR amplification efficiency prediction

**DOI:** 10.1186/1471-2105-12-404

**Published:** 2011-10-20

**Authors:** Izaskun Mallona, Julia Weiss, Egea-Cortines Marcos

**Affiliations:** 1 Genetics, Institute of Plant Biotechnology (IBV), Technical University of Cartagena (UPCT), Campus Muralla del Mar, 30202 Cartagena, Spain

## Abstract

**Background:**

Relative calculation of differential gene expression in quantitative PCR reactions requires comparison between amplification experiments that include reference genes and genes under study. Ignoring the differences between their efficiencies may lead to miscalculation of gene expression even with the same starting amount of template. Although there are several tools performing PCR primer design, there is no tool available that predicts PCR efficiency for a given amplicon and primer pair.

**Results:**

We have used a statistical approach based on 90 primer pair combinations amplifying templates from bacteria, yeast, plants and humans, ranging in size between 74 and 907 bp to identify the parameters that affect PCR efficiency. We developed a generalized additive model fitting the data and constructed an open source Web interface that allows the obtention of oligonucleotides optimized for PCR with predicted amplification efficiencies starting from a given sequence.

**Conclusions:**

pcrEfficiency provides an easy-to-use web interface allowing the prediction of PCR efficiencies prior to web lab experiments thus easing quantitative real-time PCR set-up. A web-based service as well the source code are provided freely at http://srvgen.upct.es/efficiency.html under the GPL v2 license.

## Background

Since the development of quantitative PCR (Q-PCR) in the early nineties [1], it has become an increasingly important method for gene expression quantification. Its aim is to amplify a specific DNA sequence under monitoring and measuring conditions that allow stepwise quantification of product accumulation. Product quantification has fostered the development of analysis techniques and tools. These data mining strategies focus on the cycle in which fluorescence reaches a defined threshold (value called *quantification cycle *or *Cq*) [2,3]; with the *Cq *parameter, quantification could be addressed following two approaches: (i) the standard curve method [4] and (ii) the ΔΔ*Cq *method [5].

It is worth noting that these classical quantification methods assume that amplification efficiency is constant or even equal to 100%. An efficiency value of 100% implies that during the exponential phase of the Q-PCR reaction, two copies are generated from every available template. But it has been shown that these assumptions are not supported by experimental evidences [6]. With the aim of estimating PCR efficiency, and thus to include it in further analysis procedures, two strategies have been developed: (i) kinetics-based calculation and (ii) standard curve assessment.

Taking into account the reaction kinetics, which is basically equivalent to the bacterial growth formulae [7], amplification efficiency could be visualized in a half-logarithmic plot in which log transformed fluorescence values are plotted against the time (cycle number). In these type of graphic representations, the phase of exponential amplification is linear and the slope of this line is the reaction efficiency [8]. Empirical determinations of amplification efficiencies show that ranges lay between 1.65 and 1.90 (65% and 90%) [9]. Standard curve-based calculation method relies on repeating the PCR reaction with known amounts of template. *Cq *values *versus *template (i.e. reverse transcribed total RNA) concentration input are plotted to calculate the slope. Laboratories where few genes are analyzed for diagnostic may develop standard curves but they are in most cases out of scope for research projects where tens-hundreds of genes will be tested for changes in gene expression.

Several aspects influence PCR yield and specificity: reagents concentration, primer and amplicon length, template and primer secondary structure, or G+C content [10]. The goals of a PCR assay design are: (i) obtaining the desired product without mispriming and (ii) rising yield towards optimum. In most cases, the sequence to amplify is a fixed entity, so setting up an efficient reaction involves changes in reagents concentrations (salts, primers, enzyme) and specifically an optimal primer design. Thus a plethora of primer designing tools have been published, regarding as little as G+C content for *T_m _*calculation [11,12], evaluating salt composition [13] or even employing Nearest Neighbor modules, which consider primer and salt concentrations [14].

Efficiency values are essential elements in the ΔΔ*Cq *method and its variants: relative quantities are calculated using the efficiency value as the base in an exponential equation in which the exponent depends on the *Cq*. Thus efficiency strongly influences the relative quantities calculation, which are required to estimate gene expression ratios [5].

In this work, we analysed Q-PCR efficiency values from roughly 4,000 single PCR runs with the aim of elucidating the major variables involved in PCR efficiency. With this data we developed a generalized additive model (GAM), which relies on nonlinear regression analysis, and implemented it in a open, free online web tool allowing efficiency prediction.

## Results

### Data overview

**Figure 1:**
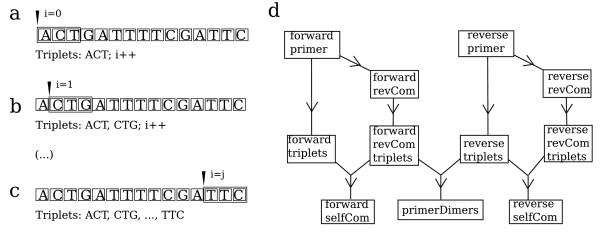
**Algorithms for primer self-complementarity (primerSelfcom) and cross hybridization (primerDimers) computing**. **(a)**, **(b) **and **(c) **show three stages of the sliding window triplet extraction step. All the DNA string is reduced into overlapping triplets. **(d) **reflects the general overview of the algorithm. As a first step, triplets are extracted for each primer. Then, primers are reverse complemented and thereafter splitted into overlapping triplets. Comparison between triplets allows the generation of an estimate of similarity, which is employed as a hybridization predictor.

Our data were generated from 90 different amplification products that included four *Escherichia coli *strains, three *Agrobacterium tumefaciens *strains [15], three tomato varieties [16], three *Petunia hybrida *lines [17], one *Antirrhinum majus *line [18], one *Opuntia ficus-indica *genotype [19] and human liquid cytology samples used to test for *Prostate Serum Antigen *presence. Efficiencies ranged between 1 (no amplification) and 2 (perfect exponential duplication). We wrote an R script (see Materials and Methods) to extract the inputs for further statistical analysis. The script regarded complete amplicon length, primer sequence, G+C content of amplicon and primers, presence of repetitions in the amplicon (N6 or above), primer melting temperature and the 3' terminal, last two nucleotides of each primer and primers tendency to hybridize (Figure 1). Metadata for each PCR reaction included sample origin (i.e. genomic or cDNA), operator involved, species and line or variety, exact sequence amplified, primer length and PCR efficiency; a summary of the data is shown as Additional File 1.

Efficiency dataset had highly repetitive data (presence of ties). As some of our comparisons intended to assess relationships between two quantitative variables, we applied Spearman tests. However, analysis of quantitative versus qualitative data was performed employing Kruskal-Wallis tests. Due to the presence of ties, which hampers the application of rank-based tests, asymptotic tests were applied. p-values were approximated *via *its asymptotic distribution and ties were adjusted *via *random rank averaging. Both procedures are implemented in the coin R package [20]. We chose a value of 0.05 as cut-off of statistical signification. A summary is shown in Table 1. *Post-hoc *asymptotic Wilcoxon Mann-Whitney rank sum test were performed to discriminate the contribution of some of the categorical variables, as well to analyzed effect sizes; the statistical outputs are shown as Additional File 2.

**Table 1:** Statistical results for univariate analysis

Univariate analysis						
**Variable**	**value**	**df**	***ρ***	**d**	**Log Odds**	**p-value**

Primers length	Z = -7.4398	-	-0.118	-0.239	-0.433	1.008e-13*
Sequence length	Z = -5.423	-	-0.086	-0.173	-0.314	5.86e-08*
Sequence G+C content	Z = -10.2664	-	-0.163	-0.331	-0.601	<2.2e-16*
A repeats	Z = 2.1004	-	0.033	0.067	0.121	0.03569*
T repeats	Z = 3.9818	-	0.063	0.127	0.230	6.84e-05*
C repeats	Z = -5.294	-	-0.084	-0.169	-0.307	1.196e-07*
G repeats	Z = -7.1808	-	-0.114	-0.230	-0.418	6.929e-13*
Primers *T_m_*	Z = 1.4653	-	0.023	0.047	0.085	0.1428
Primers self complementarity	Z = 11.9002	-	0.190	0.386	0.700	<2.2e-16*
Primer dimers	Z = 4.4161	-	0.070	0.141	0.256	1.005e-05*
Primer GC imbalance	Z = 11.1367	-	0.177	0.360	0.654	<2.2e-16*
Primers GC content	Z = 4.5921	-	0.073	0.147	0.266	4.388e-06*
Sequence palindromes	Z = -3.4951	-	-0.056	-0.111	-0.202	0.0004738*
Species	*χ*^2 ^= 585.616	9	-	-	-	<2.2e-16*
Template	*χ *^2 ^= 1241.562	12	-	-	-	<2.2e-16*
Variety or line	*χ *^2 ^= 585.8386	18	-	-	-	<2.2e-16*
Template source	*χ *^2 ^= 940.8915	24	-	-	-	<2.2e-16*
Operator	*χ *^2 ^= 727.4887	8	-	-	-	<2.2e-16*
Primer's 3' last two nucleotides	*χ *^2 ^= 237.911	15	-	-	-	<2.2e-16*

In the case of asymptotic Spearman tests, *Z *value are shown; *χ*^2 ^value is written for asymptotic Kruskal-Wallis tests. An asterisk over the p-value reflects a significant influence (p-value ≤ 0.05). Sperman's correlation analysis shows the *ρ *statistic as well the Cohen's *d *and *Log Odds *estimates of effect size. For *post-hoc *asymptotic Wilcoxon Mann-Whitney rank sum tests and its effect size estimators see Additional File 2.

**Figure 2:**
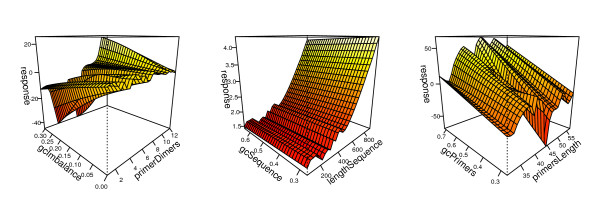
**Perspective plot views of the GAM**. Results of the best-fitting smooths for the variables included in the model. The interaction between the two variables is presented as a surface; the z-axis shows the response and the relative importance of each variable is presented in the x- and y- axis.

In order to build up a predictive statistical model we used a generalized additive modelling procedure as an effective technique for conducting nonlinear regression analysis in which factors were modeled using nonparametric smooth functions. GAM function was implemented by the R project mgcv package [21], according to the formulation described in [22]. Data fitting is shown in Table 2. Figure 2 shows perspective plot views of the GAM; predicted efficiency is plotted as a response surface defined by the values of two interacting variables.

**Table 2:** GAM overview

GAM analysis				
	**Estimate**	**Std. Error**	**t value**	***Pr*(> |*t*|)**

(Intercept)	1.73825	0.00153	1136	<2e-16

Approximate significance of smooth terms				

	edf	Ref.df	F	p-value

s(lengthSequence, gcSequence)	17.58	18.08	2.332	0.00114

s(primersLength, gcPrimers)	27.96	28.46	22.950	< 2e-16

s(gcImbalance, primerDimers)	28.83	29.33	16.717	< 2e-16

R-sq.(adj) = 0.41 Deviance explained = 42.1% GCV score = 0.0094091 Scale est. = 0.0092293 n = 3944				

GAM summary as estimated by the gam function of the mgcv R package. Model formula corresponds to: *efficiency s*(*lengthSequence*, *gcSequence*) + *s*(*primersLength*, *gcPrimers*) + *s*(*gcImbalance*, *primerDimers*).

### Statistical modelling

**Figure 3:**
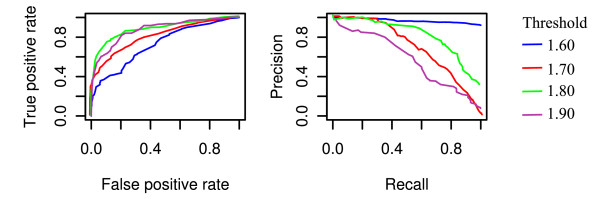
**ROC and PR curves**. ROC and PR curves are plotted for various experimental efficiency thresholds, which define the decision criteria of succesful PCR performance. A good behaviour in ROC space is to be in the upper-left-hand corner, whereas in PR space the goal is to locate at the upper-right-hand corner.

Model selection was performed according to the Akaike's Information Criterion, a penalized log-likelihood system addressing model goodness and denying low parsimony [23]. We selected a GAM based on the interaction of the length and G+C content of the sequence as well, independently, of the primers; and the interaction of the G+C imbalance between primers with an estimation of the tendency to produce primer dimers. The R squared parameter of the model is 0.41, whereas the deviance explained is 42.1%. As the model intends to estimate the PCR efficiency of a set composed by a given amplicon and a given set of oligo primer pairs, it could be validated in terms of ranking performance. We defined a threshold of experimental efficiency measured during the Q-PCR obtaining a decision criterion of adequate PCR performance. We took results below 1.65 (i.e. 65%) of efficiency as fails, thus this threshold acts as a binary classifier of success. Receiver Operator Characteristic (ROC) curves are commonly used to analyze how the number of correctly classified positive cases whose predicted efficiency is over the threshold change with the number of incorrectly classified negative examples whose predicted efficiency is below that threshold [24]. This representation is complemented with the precision and recall (PR) curves, which evaluate the relationship between precision (the ability of presenting only relevant items) and recall (true positive rate) [24]. ROC and PR curves are shown in Figure 3; ROC rises rapidly to the the upper-left-hand corner thus reflecting that the false-positive and false-negative rates are low, whereas the PR curve locates at the upper-right-hand corner and thereafter indicates that most of the items classified as positive are true positives.

### Implementation

In order to ease PCR efficiency prediction prior to wet-lab PCR set-up, we developed a user-friendly, freely available web tool for assessing primer suitability before and during primer design. For this purpose, we wrote a set of Python/Biopython scripts [25] requested through a Common Gateway Interface (CGI). These scripts were developed to call to the Primer3 software [26], working inside the EMBOSS package [27]. The web tool called PCR efficiency calculator allows primer design starting from a DNA fragment producing a set of theoretical PCR efficiency values. It also predicts PCR efficiency values for preexisting primers and DNA template combinations.

## Discussion

Several tools have been developed to assess primer design procedures. Most of them consider hairpin structure formation avoidance, selection of nucleotides in 3' *termini*, primer melting temperature, etc. [28]. However, intrinsic amplicon characteristics are not contemplated in primer design. The work we present includes this important parameter as amplification was found to be highly dependent on template structure (Tables 1 and 2). Indeed PCR specificity or PCR failure have been found to be dependent on sequence similarity between primers and template, lack of mismatches, or number of priming sites [29,30]. Using logistic regression analysis, Benita *et al. *found that PCR success is highly dependent on regionalized G+C content in the template thus showing the importance of template structure as a second step in PCR optimization. Generally, PCR success is evaluated as a dichotomy by presence or absence of product. However in Q-PCR experiments amplification efficiency becomes an important parameter to perform proper statistical analysis that should yield the actual differences in expression between several transcripts. Thus PCR efficiency becomes as important as *Cq *values to determine differential gene expression. Our model showed that G+C content in the amplicon plays a key role in PCR efficiency confirming previous work and including it inside as a predictor of PCR efficiency.

Multiple parameters, such sequence palindrome abundance or the nucleotide at the 3' primer *termini*, were found to significantly contribute to the PCR efficiency when analyzed separately. However, they were not significant when included to a multiple component GAM. Unexpectedly, variables such the species or the line the template was extracted from, or the operator involved in the PCR set-up, were found to be significant. Nonetheless, the modelling procedure disregarded these variables, as GAM with and without them did not differ significantly. Thus we ascribe this result as covariation, because each primer pair-amplicon combination is normally used in a certain project which is limited to a species, operator and one or a few lines.

The model presented in this work estimates an efficiency value *per *PCR reaction regarding three parameters, each of one represents the interaction between two independent variables: the interaction between G+C content of the amplicon with its length; the interaction between G+C content of the primers with their length; and the interaction between G+C content imbalance between primers (gcImbalance; their difference in G+C) with their tendency to hybridize and thus to form primer dimers (primerDimers). Our model gives a high influence to the difference of G+C content between primers. Previous works noted that PCR using unequal primer concentrations have better efficiencies when their melting temperatures differ in ≥ 5°C [31]. However, when our tool is piped to the Primer3 primer-design work flow, this difference is restricted by the Primer3 algorithms, thus avoiding design of highly unequal primers. Very high or very low amplicon G+C content affects amplification success [32,33]. Specially, regionalized G+C-content has been shown to be relevant in PCR success prediction [34].

The comparison of the model performance in the ROC space discriminates 1.60 as the classifier threshold which leads to the worst model behaviour, but shows only minor differences for the other cut-offs. The analysis of PR curves allows further comparisons and highlights that 1.80 shows the highest degree of resolution. Tuomi and coworkers described 1.80 as boundary for optimized PCR reactions [35].

It is worth noting that the tool developed aids in primer design prior to the wet lab experiments. Since it remains clear that there are physical constraints which establish the maximum PCR efficiency of a given set of one amplicon and a pair of oligos, bias is introduced in many ways (pipetting, reactives, PCR machine, etc.). We would like to point out that our work does not intend to substitute the experimental efficiency calculation nor modify the quantification settings; its aim is to complement the existing primer design tools and thus minimize the need for primer combination testing.

## Conclusions

Using a wide range of amplicons and PCR set-ups, we statistically modelized the response of the PCR efficiency value, a parameter affecting PCR success and involved in effective gene expression quantitation. In order to ease PCR primer design for Q-PCR experiments, the efficiency-predicting model was included in the Primer3 design pipeline and freely provided as a web tool. This tool should help to generate primer combinations with similar theoretical efficiencies to well established PCR primers or to ease multiplex PCR reactions where efficiencies should be similar among templates.

## Methods

### DNA templates

We used a variety of DNA templates to obtain data for efficiencies including genomic DNA from bacteria, yeasts, plants and humans, and plasmid DNA. Samples using cDNA as template were produced from isolated mRNA from different sources. Synthesis of first strand cDNA was performed from DNAase treated mRNA, using the Maxima kit from Fermentas as described in the protocol. Samples amplified by whole genome amplification using the *ϕ*29 DNA polymerase were performed with the Genomiphi kit (GE-Healthcare) according to manufacturers manual. A summary of the data is available as Additional File 1.

### Real time PCR

PCR reactions used were carried out with the SYBR Premix Ex Taq (TaKaRa Biotechnology, Dalian, Jiangsu, China) in a Rotor-Gene 2000 thermocycler (Corbett Research, Sydney, Australia) and analysed with Rotor-Gene analysis software v.6.0 as described before [17]. A second set of reactions was performed with a Mx3000P machine (Stratagene, Amsterdam) and analyzed with the qpcR R package [36]. Reaction profiles used were 40 cycles at 95°C for 30 s, an amplicon-specific annealing temperature for 20 s and amplification at 72°C. In order to ensure the specificity of the reaction, uniqueness of Q-PCR products were checked by melting analysis (data not shown). Fluorescence data acquisitions during the cycling steps were collected at 72°C step, temperature at which eventual primer-dimers should be melted, thus avoiding artifactual contribution to the fluorescence measure. Once finished, analysis was followed by a melting curve whose ramp was delimited between the annealing temperature and 95°C. Reaction volume was 15 *μ*L and each primer was 240 nM.

Reaction efficiency was calculated using the amplification curve fluorescence, analyzing each PCR reaction (tube) separately as before [37]. Efficiency value (*E*) was defined as $E=\frac{F_{n}}{F_{n}-1}$, in which *n *is determined as the 20% value of the fluorescence at the maximum of the second derivative curve. Efficiency calculations were performed with the qpcR R package [36]. Curves were formed by 40 points, each one representing a fluorescence measure in each amplification cycle. The Rotor-Gene 2000-based runs were baseline corrected either by standard normalization (substraction of the fluorescence present in the first five cycles of each sample) or by "dynamic tube" normalization (which uses the second derivative of each sample trace to determine the take-off, thus asigning a threshold separately to each reaction), whereas the Mx3000P were by "adaptive baseline" correction (which assigns a threshold independently to each sample).

### Data mining

Data mining was performed with the R statistical environment v2.7.1 and v2.10.1 [38] with the following libraries: coin v1.0-4 [20], mgcv v1.7-5 [21], ROCR [39] v1.0-4, compute.es v0.2 [40] and verification v1.31 [41]. The final model was implemented in a CGI server-side set of Python/Biopython scripts interacting with the web browser requests. Source code of both modelling procedures and the server-side application are available at the website.

## Authors' contributions

IM, ME and JW carried out the design of the study. IM and JW performed the Q-PCR experiments and participated in data collection. IM designed the algorithms, developed the model and programmed the application. IM and ME wrote the manuscript. All authors read and approved the final manuscript.

## Supplementary Material

Additional file 1 **Data overview**. Data comprise 90 different amplification products that included different template sources. Efficiencies ranged between 1 (no amplification) and 2 (perfect exponential duplication). Measured parameters contained: complete amplicon length (*lengthSequence*), primer length (*forLength *and *revLength*, for the forward and reverse primers respectively), G+C content of amplicon and primers (*gcSequence *and *gcPrimers*), logical variables showing the presence of N6 or above repeats (*aRepeats, tRepeats, cRepeats, gRepeats*); and *aCount, tCount, cCount, gCount *integer variables regarding the length of the longest repeat found), primer melting temperature (*tmForward*, *tmReverse*), tendency to form primer dimers (*primerDimers*), tendency to selfcomplementarity (*primersSelfcom*), and the 3' terminal two nucleotides of each primer (*trap3For, trap3Rev*) as well the terminal last nucleotides of each primer (*trap3LastFor, trap3LastRev*). Metadata for each PCR reaction included the thermocycler used (*machine*), sample origin (i.e. genomic or cDNA; *template*) and organ involved (*source*), person involved (*operator*), species (*species*) and line or variety (*var*), presence of palindromes at the amplicon (*sequencePalindromes*), primer length (*primersLength*), and PCR efficiency (*efficiency*). Click here for file

Additional file 2 **Post-hoc categorical variable analysis**. The variables regarding PCR template (*GD*, genomic; *CD*, cDNA; *plasmid*, *Escherichia coli *plasmid; *yGD*, yeast genomic) and 3' primer *termini *(*U*, purine; *Y*, pyrimidine) were analyzed by asymptotic Wilcoxon Mann-Whitney rank sum tests (*Z*, Z value; *p*, p-value), and the effect sizes estimated by the two tailed p-value (Cohen's *d*, mean difference; Hedge's *g*, unbiased estimate of *d*; *r*, correlation coefficient; and *n*, the total sample size, which is twice the effective sample size when the *termini *of the two oligos are analyzed). Click here for file
