# Supplementary material for: pcrEfficiency: a Web tool for PCR amplification efficiency prediction
Source: BMC Bioinformatics. 2011 Oct 20;12:404. doi: 10.1186/1471-2105-12-404 (PMC3234296; doi:10.1186/1471-2105-12-404)
Supplement: Additional file 1 — Data overview. Data comprise 90 different amplification products that included different template sources. Efficiencies ranged between 1 (no amplification) and 2 (perfect exponential duplication). Measured parameters contained: complete amplicon length (lengthSequence), primer length (forLength and revLength, for the forward and reverse primers respectively), G+C content of amplicon and primers (gcSequence and gcPrimers), logical variables showing the presence of N6 or above repeats (aRepeats, tRepeats, cRepeats, gRepeats); and aCount, tCount, cCount, gCount integer variables regarding the length of the longest repeat found), primer melting temperature (tmForward, tmReverse), tendency to form primer dimers (primerDimers), tendency to selfcomplementarity (primersSelfcom), and the 3' terminal two nucleotides of each primer (trap3For, trap3Rev) as well the terminal last nucleotides of each primer (trap3LastFor, trap3LastRev). Metadata for each PCR reaction included the thermocycler used (machine), sample origin (i.e. genomic or cDNA; template) and organ involved (source), person involved (operator), species (species) and line or variety (var), presence of palindromes at the amplicon (sequencePalindromes), primer length (primersLength), and PCR efficiency (efficiency). [file 1471-2105-12-404-S1.PDF]

Supplemental table 1

Table 1: Data overview. Data comprises 90 different amplification products that included different template sources. Efficiencies ranged between 1 (no amplification) and 2 (perfect exponential duplication). Measured parameters contained: complete amplicon length (*lengthSequence*), primer length (*forLength* and *revLength*, for the forward and reverse primers respectively), G+C content of amplicon and primers (*gcSequence* and *gcPrimers*), presence of repetitions in the amplicon (*aRepeats, tRepeats, cRepeats, gRepeats* logical variables showing the presence of N6 or above repeats; and *aCount, tCount, cCount, gCount* as integer variables regarding the lenght of the longest repeat found), primer melting temperature (*tmForward*, *tmReverse*), tendency to form primer dimers (*primerDimers*), tendency to selfcomplementarity (*primersSelfcom*), and the 3' terminal two nucleotides of each primer (*trap3For, trap3Rev*) as well the terminal last nucleotides of each primer (*trap3LastFor, trap3LastRev*). Metadata for each PCR reaction included the thermocycler used (*machine*), sample origin (i.e. genomic or cDNA; *template*) and organ involved (*source*), person involved (*operator*), species (*species*) and line or variety (*var*), presence of palindromes at the amplicon (*sequencePalindromes*), primer length (*primersLength), and PCR efficiency (efficiency).*

	species	name	forLength	revLength	template
1	P.hybrida :1795	PhChS : 240	Min. :15.00	Min. :14.00	CD :2121
2	A.majus : 989	AmRan3 : 195	1st Qu.:20.00	1st Qu.:20.00	GD :1358
3	S.lycopersicum: 459	AmHistone4: 185	Median :20.00	Median :20.00	GN : 98
4	O.ficus-indica: 369	PhCypP450 : 159	Mean :20.48	Mean :20.58	genomiphi: 58
5	E.coli : 114	AmCyclinA : 157	3rd Qu.:21.00	3rd Qu.:21.00	CF : 57
6	S.cerevisiae : 67	PhPmads : 155	Max. :27.00	Max. :31.00	plasmid : 55
7	(Other) : 151	(Other) :2853			(Other) : 197

	efficiency	source	operator	var	machine
1	Min. :1.010	Petal :561	Julia :756	V30 :782	rotor :1545
2	1st Qu.:1.670	Leaf :533	Izaskun :728	T2 :471	stratagene: 858
3	Median :1.720	Fruit :314	Mara :470	Mitchell:419	
4	Mean :1.735	Cell :122	JuanFran :320	Microtom:309	
5	3rd Qu.:1.810	FlowerA:121	Magali :102	Top10 :106	
6	Max. :2.000	Library:110	Agniezska: 27	T1 : 76	
7		(Other):642	(Other) : 0	(Other) :240	

	lengthSequence	primersLength	forward	reverseRevcom	reverse
1	Min. : 74.0	Min. :31.00	Length:3944	Length:3944	Length:3944
2	1st Qu.:114.0	1st Qu.:40.00	Class :character	Class :character	Class :character
3	Median :190.0	Median :40.00	Mode :character	Mode :character	Mode :character
4	Mean :246.1	Mean :41.07			
5	3rd Qu.:284.0	3rd Qu.:41.00			
6	Max. :907.0	Max. :57.00			

	gcSequence	gcPrimers	gcImbalance	aRepeats	tRepeats
1	Min. :0.2691	Min. :0.2702	Min. :0.00000	Length:3944	Length:3944
2	1st Qu.:0.4148	1st Qu.:0.4631	1st Qu.:0.02381	Class :character	Class :character
3	Median :0.4560	Median :0.4643	Median :0.02619	Mode :character	Mode :character
4	Mean :0.4591	Mean :0.4842	Mean :0.05625		
5	3rd Qu.:0.5049	3rd Qu.:0.5119	3rd Qu.:0.08095		
6	Max. :0.6486	Max. :0.7171	Max. :0.30326		

	cRepeats	gRepeats	aCount	tCount	cCount
1	Length:3944	Length:3944	Min. :0.000	Min. : 0.0000	Min. :0.000
2	Class :character	Class :character	1st Qu.:0.000	1st Qu.: 0.0000	1st Qu.:0.000
3	Mode :character	Mode :character	Median :1.000	Median : 0.0000	Median :0.000
4			Mean :1.357	Mean : 0.8945	Mean :0.426
5			3rd Qu.:2.000	3rd Qu.: 1.0000	3rd Qu.:1.000
6			Max. :8.000	Max. :16.0000	Max. :3.000

	gCount	tmForward	tmReverse	tmPrimers	trap3For
1	Min. :0.0000	Min. :44.64	Min. :40.69	Min. :43.16	Length:3944
2	1st Qu.:0.0000	1st Qu.:49.73	1st Qu.:52.40	1st Qu.:51.07	Class :character
3	Median :0.0000	Median :51.78	Median :52.97	Median :52.69	Mode :character
4	Mean :0.5228	Mean :51.70	Mean :53.29	Mean :52.49	
5	3rd Qu.:1.0000	3rd Qu.:54.36	3rd Qu.:56.04	3rd Qu.:54.36	
6	Max. :5.0000	Max. :59.98	Max. :60.80	Max. :59.12	

	trap3Rev	trap3LastFor	trap3LastRev	forwardRevcom	forwardSelfcom
1	Length:3944	Length:3944	Length:3944	Length:3944	Min. : 0.000
2	Class :character	Class :character	Class :character	Class :character	1st Qu.: 2.000
3	Mode :character	Mode :character	Mode :character	Mode :character	Median : 5.000
4					Mean : 4.136
5					3rd Qu.: 6.000
6					Max. :10.000

	reverseSelfcom	primersSelfcom	primerDimers	sequencePalindromes
1	Min. : 0.000	Min. : 0.000	Min. : 1.000	Min. : 0.000
2	1st Qu.: 2.000	1st Qu.: 6.000	1st Qu.: 4.000	1st Qu.: 0.000
3	Median : 3.000	Median : 8.000	Median : 6.000	Median : 1.000
4	Mean : 4.386	Mean : 8.522	Mean : 5.426	Mean : 1.969
5	3rd Qu.: 7.000	3rd Qu.:11.000	3rd Qu.: 7.000	3rd Qu.: 3.000
6	Max. :13.000	Max. :18.000	Max. :12.000	Max. :10.000
