# Supplementary material for: pcrEfficiency: a Web tool for PCR amplification efficiency prediction
Source: BMC Bioinformatics. 2011 Oct 20;12:404. doi: 10.1186/1471-2105-12-404 (PMC3234296; doi:10.1186/1471-2105-12-404)
Supplement: Additional file 2 — Post-hoc categorical variable analysis. The variables regarding PCR template (GD, genomic; CD, cDNA; plasmid, Escherichia coli plasmid; yGD, yeast genomic) and 3' primer termini (U, purine; Y, pyrimidine) were analyzed by asymptotic Wilcoxon Mann-Whitney rank sum tests (Z, Z value; p, p-value), and the effect sizes estimated by the two tailed p-value (Cohen's d, mean difference; Hedge's g, unbiased estimate of d; r, correlation coefficient; and n, the total sample size, which is twice the effective sample size when the termini of the two oligos are analyzed). [file 1471-2105-12-404-S2.PDF]

Supplemental table 2

Table 1: Post-hoc categorical variable analysis. The variables regarding PCR template (*GD*, genomic; *CD*, cDNA; *plasmid*, *Escherichia coli* plasmid; *yGD*, yeast genomic) and 3' primer *termini* (*U*, purine; *Y*, pyrimidine) were analyzed by asymptotic Wilcoxon Mann-Whitney rank sum tests (Z, Z value; p, p-value), and the effect sizes estimated by the two tailed p-value (d, mean difference; g, unbiased estimate of d; r, correlation coefficient; and n, the total sample size (which is twice the effective sample size when the *termini* of the two oligos are analyzed).

Template	Z	p-value	d	g	r	n
CD *vs.* GN	-31.1635	<2.2e-16	0.271137920	0.271085084	0.1317235345	3851
CD *vs.* plasmid	-12.0695	<2.2e-16	0.559512222	0.559347385	0.1615837128	2548
GD *vs.* plasmid	-9.9228	<2.2e-16	0.574095802	0.573854281	0.1925205081	1785
yGD *vs.* plasmid	8.8896	<2.2e-16	0.777071206	0.775851314	0.362157469	480
yGD *vs.* CD	3.9596	7.506e-05	0.269510332	0.269430870	0.078361414	2546
yGD *vs.* GD	9.7282	<2.2e-16	0.576176063	0.575933395	0.1926264601	1783
UU *vs.* YY	-8.3872	<2.2e-16	0.275348845	0.275291444	0.1361974158	3600
UU *vs.* UY	-6.2664	3.695e-10	0.1977330262	0.1976975202	0.0967138293	4179
UU *vs.* YU	-8.741	<2.2e-16	0.278347520	0.278288124	0.1377802172	3517
UY *vs.* YU	-3.1566	0.001596	0.0976434970	0.0976264096	0.0481803989	4288
UY *vs.* YY	-2.2721	0.02308	0.069369048	0.069357139	0.034358580	4371
YU *vs.* UY	-3.1566	0.001596	0.0976434970	0.0976264096	0.0481803989	4288
